# Comparing the shear bond strength of veneering materials to the PAEKs after surface treatments

**DOI:** 10.1186/s12903-023-02879-2

**Published:** 2023-03-30

**Authors:** Mustafa Kiliç, Doğu Ömür Dede, Ahmet Serkan Küçükekenci

**Affiliations:** 1 Graduate Prosthodontics, ADC Alfa Oral and Dental Health Center, Istanbul, Turkey; 2 Associate Professor, Department of Prosthodontics, Faculty of Dentistry, Ordu University, 52200 Altınordu/Ordu, Turkey

**Keywords:** PEEK, PEKK, Surface treatment, Shear bond strength, Veneering material

## Abstract

**Background:**

This study aimed to evaluate the impact of various surface treatments on the shear bond strength (SBS) of polyetheretherketone (PEEK) and polyetherketoneketone (PEKK) polymers to indirect laboratory composite (ILC) and lithium disilicate ceramic (LDC) veneering materials.

**Methods:**

Polymer specimens (7 × 7x2 mm) were sectioned from PEEK and PEKK discs (*N* = 294) and randomly allocated to 7 groups (*n* = 20); untreated (Cnt), plasma (Pls), 98% sulfuric acid (Sa), sandblasting with 110 µm Al_2_O_3_ (Sb), tribochemical silica coating with 110 µm silica modified Al_2_O_3_ (Tbc), Sb + Sa, Tbc + Sa. Scanning electron microscopy assessments were performed on one sample of each treatment group, and veneering materials were applied to the remaining specimens (*n* = 10). The specimens were subjected to the SBS test after being soaked in distilled water (24 h, 37 °C). Three-way ANOVA, independent sample t-test, and Tukey HSD test were performed for statistical analyses (α = .05).

**Results:**

The surface treatment, polymer, veneering material types, and their interactions were significant on SBS results according to the 3-way ANOVA (*p* < 0.001). The SBS values of ILC veneered groups were significantly higher than LDC groups, regardless of surface treatment and polymer type (*p* < 0.05). The highest SBS values were obtained for Sa-applied ILC veneered PEEK (21.55 ± 1.45 MPa) and PEKK (17.04 ± 1.99 MPa) polymer groups (*p* < 0.05).

**Conclusion:**

The effect of surface treatment and veneering materials may be significant on the SBS values of PAEKs. Therefore, the application parameters of surface treatments should be more specified for the applied veneering material and polymer type.

## Introduction

Polyaryletherketones (PAEKs) are advanced high-performance thermoplastic resins that consist of aromatic benzene chains connected by functional ether or ketone groups [1–3]. While PAEK polymers are named according to the ratio of ether and ketone groups, this ratio is also responsible for the rigidity, glass conversion degree, and melting point of the polymers [4, 5]. While polyetheretherketone (PEEK) is the most popular and proven resin in the PAEK family, the recently introduced polyetherketoneketone (PEKK) polymer has a higher melting point, higher compressive strength (80%) due to the improved solidifying of glass and polymer chains with higher ketone content [3, 6]. Due to their biocompatibility and superior mechanical properties, PAEKs have been very attractive medical materials since the '90 s, such as orthopaedic implants and prosthesis manufacturing [1, 2]. The PAEKs have also been utilized in dentistry, commonly to fabricate transitional and healing abutments, dental implants, clasp, and the framework of removable partial dentures and fixed dental prostheses substructure [4, 7].

While the PAEKs have many superior features, the material's low translucent and white-greyish colour limited the monolithic usage for fixed partial restorations, especially in the esthetic region [8]. For this reason, PAEKs substructures have been commonly veneered with di-methacrylate (DMA) or methyl methacrylate (MMA) based resin materials and with ceramic-based restoratives to gain acceptable esthetic results [8, 9]. The indirect laboratory composite (ILC) resin veneers are most popular due to their superior bonding performance, mechanical features, wide range of colours, easy manipulation, and repairability features, but ceramic veneers, especially lithium disilicate ceramics (LDC), have become more attractive with life-like appearance, colour stability, biocompatibility [3, 10]. However, the inert structure and low surface energy of PAEKs are still challenging to achieve sufficient bond strength with veneer materials [8, 11]. Various micromechanical and chemical surface treatment procedures have been tested on PAEKs to alter the surface characteristics for more durable and better bonding, including sandblasting(Sb) with aluminium oxide (Al_2_O_3_) [12], tribochemical silica coating (Tbc) with silica (SiO_x_) modified Al_2_O_3_ [3], etching with sulfuric acid (Sa) or its mixture with hydrogen peroxide (Piranha solution) [9, 13], plasma application [14, 15], and laser applications [16]. The etching with Sa solution, especially in the concentration of 98%, was the most effective surface treatment technique on PEEK polymers [8, 11, 13, 17], and favourable bonding results have also been declared for Sb and Tbc techniques on both PEEK and PEKK polymers [3, 16].

The plasma treatment, which mainly consists of generated ionized gas particles under a high electric field, was shown as an alternative or supporting surface treatment procedure on PAEKs. The plasma may be applied with-without heat release (thermal or cold), pressure, or gas supply (oxygen, nitrogen, argon), according to the used system [15, 18]. A cold atmospheric plasma (Pls) system with hand-held devices may be used to increase the higher bond strength of polymers to resin materials by increasing the wettability and surface free energy [5, 15, 19]. A bonding agent application is also essential for the chemical conditioning of inert polymer materials before the veneering and cementation protocols. A functional monomer containing a bonding agent and following opaquer application to PAEKs has been reported in many studies to increase the bonding values [10, 19, 20]. The bonding agents with DMA, MMA, or pentaerythritol tri-acrylate (PETIA) ingredients have been correlated to the higher bond strength results on PAEKs in the previous studies [21, 22].

The surface treatments with micro-mechanical and chemical conditioning techniques that impact the bond strength of PEEK to veneer materials have been investigated in previous studies [8, 16, 20]. Still, limited information is available on the recently introduced PEKK polymer [3, 10]. Therefore, the present surface treatment techniques on the PEKK were performed according to the manufacturer's recommendations and the adapted information of PEEK polymer [3]. Additionally, the lack of information about the bonding performance of LDC veneers to PAEKs must be eliminated. This performance should also be compared with the frequently used ILC veneer materials. Thus, the present study aimed to evaluate the influence of different surface treatments on the SBS of PAEKs on ILC and LDC veneering materials. The null hypothesis was that the surface treatments would not improve the SBS of PAEKs to veneering materials, and the type of polymer and veneering materials could not vary the SBS results.

## Materials and methods

The materials and equipment used in this study are listed in Table 1. Two hundred ninety-four rectangular (7 × 7x2 mm) PEEK and PEKK specimens were sectioned from PEEK (CopraPeek; CopraPeek, Whitepeaks Dental Solutions GmbH & Co. KG, Essen, Germany) and PEKK (Pekkton ivory; Cendres + Métaux SA, Biel/Bienne, Switzerland) polymer discs using a precision saw (Mecatome T180; Presi Metallography, Presi, France) under water cooling [23]. The SBS specimens were ingrained into blocks of 20 × 30 mm auto-polymerizing acrylic resin (Panacryl; Arma Dental, Istanbul, Turkey). All specimens' surfaces were polished to standardization with sandpapers (600 to 1200-grit) (Atlas Waterproof Sheet; Saint-Gobain Abrasives, Atlas Ltd, Kocaeli, Turkey) under the rinsing water. The specimens were then ultrasonically cleaned (Branson 8510; Branson Ultrasonic, Danbury, CT, USA) for 8 min, and all specimens were allocated into seven groups (*n* = 21) according to the surface treatment applications using a simple randomization technique (Table 2). The surface topographies of each surface-treated PEEK and PEKK specimen were assessed with Scanning Electron Microscopy (SEM) (SU 1510; Hitachi High-Technologies Corp, Tokyo, Japan) (× 1200).

**Table 1:** Used materials and equipment in this study

Material	Product	Composition	Manufacturer	Lot No
PEEK	Copra Peek	PEEK (%100)	Whitepeaks Dental Solutions GmbH&Co KG	E10129
PEKK	Pekkton® ivory	PEKK (%80), TiO_2_ (%20)	Cendres + Metaux,	347,597
Indirect laboratory composite	Anaxblend Dentin Flow D-A2	UDMA, BDDMA, 0.7–1.5 μm glass powder, Fe_2_O_3_,TiO_2_, H_4_SiO_4,_ activators, stabilizers	Anaxdent GmbH	2,018,004,307
Lithium disilicate glass–ceramic	IPS e.max Press	SiO_2_-Li_2_O, K_2_O, ZnO, P_2_O_5_, Al_2_O_3_, ZrO_2_	Ivoclar Vivadent,	W13590
Sulfuric acid solution		%98 H_2_SO_4_	Brtr Kimya	951
Aluminum oxide particle	Cobra Aluminum Oxide Blasting Agent	110 μm Al_2_O_3_	Renfert,	
Silica-modified aluminum oxide particle	Rocatec Plus	110 μm SiO_x_-Al_2_O_3_	3 M Espe	65,471,222
Bonding agent	Visio.link	MMA, DMA, PETIA, C_9_H_8_O_3,_ activators, stabilizers	Bredent GmbH	190,902
Opaquer	Anaxblend Opaquer Paste	UDMA, BDDMA, Fe_2_O_3_, TiO_2_, H_4_SiO_4_, activators, stabilizers	Anaxdent GmbH	2,018,003,364
Dual-polymerized composite resin cement	Panavia V5	Bis-GMA, TEGDMA, MMA, filler, silicate glass, initiator	Kuraray Europe GmbH	000,079
Hydrofluoric acid gel	Porcelain Etch	%9.5 HF	Bisco Inc	1,900,006,968
Primer	Clearfil Ceramic Primer Plus	MATMS, MDP, Etanol	Kuraray Noritake	AV0049

*Al*_*2*_*O*_*3*_ Aluminium oxide, *BDDMA* 1,4-Butanediol dimethacrylate, *C*_*9*_*H*_*8*_*O*_*3*_ 2-propenoic acid, *DMA* Dimethyl acrylate, *Fe*_*2*_*O*_*3*_ Iron oxide, *HF* Hydrofluoric acid, *H*_*4*_*SiO*_*4*_ Silicic acid, *H*_*2*_*SO*_*4*_ Sulfuric acid, *K*_*2*_*O* Potassium oxide, *MATMS* 3-Methacryloxypropyl trimethoxysilane, *MDP* 10-methacryloyloxydecyl dihydrogen phosphate, *MMA* Methylmethacrylate, *NHC* Nanohybrid composite, *PEEK* Polyetheretherketone, *PEKK* Polyetherketoneketone, *PMMA* Polymethyl methacrylate, *P*_*2*_*O*_*5*_ Phosphorous pentoxide, *SiO*_*x-*_*Al*_*2*_*O*_*3*_ Silica-modified aluminum oxide, *TiO*_*2*_ Titanium dioxide, *UDMA* Urethane dimethacrylate, *ZnO* Zinc oxide, *ZrO*_*2*_ Zirconium oxide

**Table 2:** Surface treatment procedures

Surface treatment	Procedure
Control (Cnt)	Untreated
Plasma (Pls)	Atmospheric cold plasma surface treatment was applied with a plasma pen device (Piezobrush PZ2; Relyon Plasma) with 60 kh frequency and 30 W power output parameters. This procedure was applied at a 10 mm distance perpendicular to polymer specimens for 90 s
Sulfuric acid (Sa)	The surface of the polymer specimens was etched with %98 sulfuric acid solution (%98 H_2_SO_4_; Brtr Kimya) by dripping a drop of solution using a glass Pasteur Pipette. This procedure was applied to the polymer specimens for 60 s, rinsed for 60 s, and air-dried
Sandblasting (Sb)	The sandblasting procedure was applied with 110 Al_2_O_3_ particles (Cobra Aluminum; Renfert) at 2.5 bar pressure, 10 mm distance perpendicular to polymer specimens, for 10 s
Tribochemical Silica Coating (Tbc)	The tribochemical silica coating procedure was applied with 110 μm SiO_x_-Al_2_O_3_ particles (Rocatec Plus; 3 M Espe) at 2.5 bar pressure, 10 mm distance perpendicular to polymer specimens for 10 s
Sandblasting + Sulfuric acid (Sb + Sa)	The specimens were abraded as described in Group Sb and subsequently etched with %98 sulfuric acid as described in Group Sa
Tribochemical Silica Coating + Sulfuric acid (Tbc + Sa)	The specimens were tribochemical silica coated as described in Group Tbc and subsequently etched with %98 sulfuric acid as described in Group Sa

A bonding agent (Visio. link; Bredent GmbH, Senden, Germany) has been applied onto the surface of each specimen after the surface treatment procedures and light polymerized (Labolight DUO; GC Europe, GC Europe N.V., Leuven, Belgium) at 220 mW/cm^2^ for 90 s. Each surface treatment applied specimen group was then separated into equal subgroups (*n* = 10) according to the veneer material application, using simple randomization techniques; LDC (IPS e.max Press; Ivoclar Vivadent AG, Schaan, Liechtenstein) and ILC (Dentin Flow D-A2; Anaxdent GmbH, Stuttgart, Germany). The disc shape (3 × 2 mm) wax patterns (*n* = 140) were prepared, moulded, and the LDC ingots were heat-pressed using the lost-wax technique considering manufacturers' recommendations. The LDC disc specimens were polished to surface standardization with sandpapers (600 to 1200-grit) under the rinsing water. Then, the bonding surface of LDC specimens was etched with 9.5% hydrofluoric acid gel (Bisco Inc., Schaumburg, IL, USA) for 90 s, washed, air-dried, and a ceramic primer agent (Clearfil Ceramic Primer Plus; Kuraray Noritake, Tokyo, Japan) applied. Next, a thin layer of dual-cured resin cement (Panavia V5; Kuraray Europe GmbH) was applied onto the treated surface of polymer specimens. The etched surface of LDC specimens was placed over. After removing the residual cement, the resin cement was light polymerized (Valo; Ultradent Products Inc., Utah, USA) at 1000 mW/cm^2^ power output for 30 s.

Before the application of ILC veneering material, a thin opaquer layer (Anaxgum Opaquer Paste; Anaxdent GmbH, Stuttgart, Germany) was arranged onto the polymer surfaces using a polyethylene film with a circular hole (3.0 × 0.1 mm) and light polymerized (Labolight DUO) at 220 mW/cm^2^ for 90 s. Next, the disc shape (3 × 2 mm) ILC veneering specimens were prepared by incremental application over the opaque layer using a Teflon mould and light-polymerized for 90 s (*n* = 140). After the bonding procedures of veneer material groups were completed, all specimens were hydrolytically aged in a 37° distilled water bath for 24 h.

**Fig. 1:**
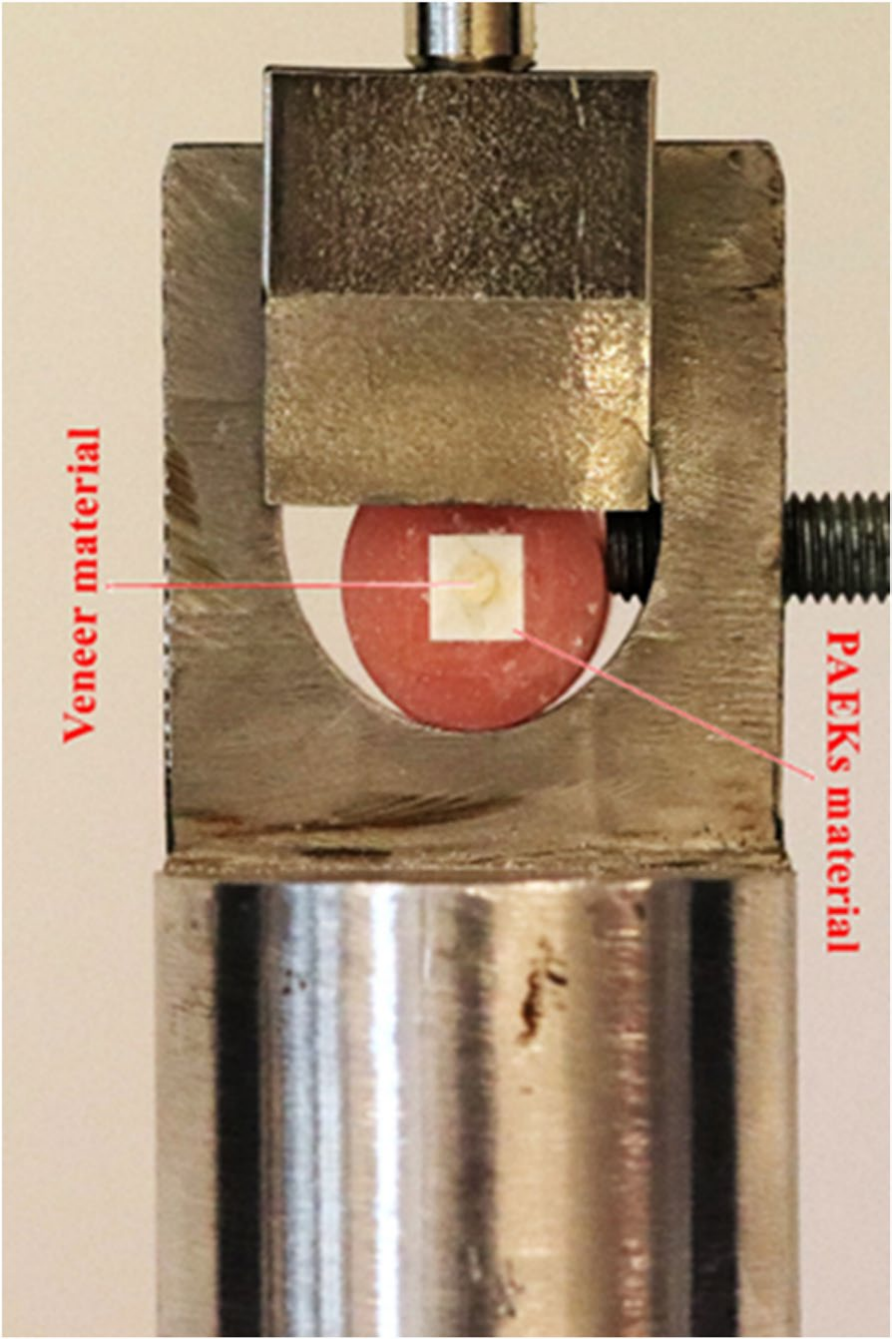
Experimental design of shear bond testing

The SBS tests have been performed using a universal test device (Autograph AGS X; Shimatsu Corp, Kyoto, Japan). While the specimen-embedded acrylic blocks were connected to the device's holder, a knife-edged shape load tip was directed to the bonding interface at a crosshead speed of 1.0 mm/min until failure occurred (Fig. 1). The maximum force value (Newton) was recorded and converted into the megapascals (MPa) by dividing the bonding surface area as described in ISO 10477 standards (α = P/A) [23]. After the SBS test, failure modes were examined under × 25 magnification with the aid of a stereomicroscope (Leica SP1600; Leica, Wetzlar, Germany) and described as adhesive (between the polymer and veneering material), cohesive (within the veneering material), and mixed (both adhesive and cohesive failures occurred).

The Kolmogorov–Smirnov and Shapiro–Wilk tests were used to evaluate the normality of data distribution and Levene's test for homogeneity. The surface treatment, polymer type, veneering material variations, and their interactions influence the SBS values, and the descriptive statistics were evaluated with the 3-way ANOVA. The mean SBS values of test groups were multiplied and compared with the Tukey HSD test and binary with independent samples t-test. The Pearson Chi-Square test was used for failure mode analyses and the Pearson correlation analyses for the correlation between the SBS and failure modes. All analyses were performed using the same statistical software program (IBM SPSS Statistics; v20.0; IBM Corp) (α = 0.05).

## Results

The 3-way ANOVA results showed that all variables and their interactions except the polymer type * surface treatment interaction (*p* = 0.653) were significant on SBS results (*p* < 0.001) (Table 3). The statistical summaries of test groups' SBS values (MPa) are listed in Tables 4 and 5, respectively.

**Table 3:** Three-way ANOVA results of SBS values

Source	Sum of Squares	df	Mean Square	F	P*
Polymer type (A)	495.415	1	495.415	172.680	.001
Veneer material (B)	1451.424	1	1451.424	505.903	.001
Surface treatment (C)	1083.976	6	180.663	62.971	.001
A x B	.583	1	.583	.203	.653
A x C	273.757	6	45.626	15.903	.001
B x C	101.654	6	16.942	5.905	.001
A x B x C	149.056	6	24.843	8.659	.001
Error	722.982	252	2.869		
Total	58,324,254	280			
Corrected Total	4251,861	279			

^*^p < 0.05 indicates a significant difference. *df* Degree of freedom, *F* F-ratio

**Table 4:** Mean and standard deviation (SD) of SBS values (MPa), and the statistical comparisons of PEEK groups

**Veneer Material**	**ILC**		**LDC**		
Surface Treatment	**Mean/ SD**	Tukey HSD*	**Mean/ SD**	Tukey HSD*	t-Test**
Cnt	11.12 ± /2.06	a	9.67 ± 1.14	a	*p* = 0.071
Pls	15.14 ± 1.23	b	10.24 ± 1.44	a	*p* < 0.001
Sa	21.55 ± 1.45	d	15.68 ± 2.09	c	*p* < 0.001
Sb	17.37 ± 1.38	bc	13.88 ± 1.77	bc	*p* < 0.001
Tbc	17.41 ± 1.21	bc	13.13 ± 1.44	b	*p* < 0.001
Sb + Sa	19.70 ± 2.22	cd	14.16 ± 1.73	bc	*p* < 0.001
Tbc + Sa	20.48 ± 2.75	d	13.49 ± 1.73	bc	*p* < 0.001

^*^Multiple comparisons between surface treatment groups for each veneer material are shown as letters, and values having the same letters are not significantly different for the Tukey HSD test (*p* > 0.05)

^**^ Pairwise comparisons of the same surface treatment applied ILC and LDC veneer materials, according to the independent sample t-Test

**Table 5:** Mean and standard deviation (SD) of SBS values (MPa) and the statistical comparisons of PEKK groups

**Veneer Material**	**ILC**		**LDC**		
Surface Treatment	**Mean/ SD**	Tukey HSD*	**Mean/ SD**	Tukey HSD*	t-Test**
Cnt	11.49 ± 1.48	a	7.57 ± 1.16	a	*p* < 0.001
Pls	15.31 ± 2.44	b	8.99 ± .73	ab	*p* < 0.001
Sa	17.04 ± 1.99	b	10.13 ± .77	bc	*p* < 0.001
Sb	16.56 ± 1.65	b	11.47 ± .97	cd	*p* < 0.001
Tbc	16.19 ± 1.83	b	11.37 ± 1.49	cd	*p* < 0.001
Sb + Sa	16.45 ± 2.39	b	11.87 ± 1.65	d	*p* < 0.001
Tbc + Sa	10.84 ± 1.27	a	10.83 ± 1.47	cd	*p* = 0.992

^*^Multiple comparisons between surface treatment groups for each veneer material are shown as letters, and values having the same letters are not significantly different for the Tukey HSD test (*p* > 0.05)

^**^ Pairwise comparisons of the same surface treatment applied ILC and LDC veneer materials, according to the independent sample t-Test

When the PEEK polymer groups were evaluated (Table 4), it was shown that all surface treatment applications (except Pls applied LDC group) significantly improved the SBS values for both veneer materials, compared to the control groups (*p* < 0.05). The highest SBS values were obtained for Sa applied to both ILC (21.55 ± 1.45 MPa) and LDC (15.68 ± 2.09 MPa) veneer groups, but not significant with the combined treatment groups (*p* > 0.05). Furthermore, no significant difference was obtained between the Sb, Tbc, and their combined treatment groups for both veneer materials (*p* > 0.05). The ILC veneer groups' SBS values were significantly higher than LDC groups for all surface treatments (except Cnt) (*p* < 0.001).

When the PEKK polymer groups were evaluated (Table 5), the SBS values of ILC veneer groups were significantly higher than LDC groups for all surface treatments (except Tbc + Sa) (*p* < 0.001). No significant improvement was determined for the SBS values of Tbc + Sa applied ILC (10.84 ± 1.27 MPa) and Pls applied LDC (10.84 ± 1.27 MPa) groups, compared to the control groups (*p* > 0.05). The SBS values of the remaining ILC groups were higher than the control group *(p* < 0.05), with no significant difference among them (*p* > 0.05). The highest SBS values were achieved for the Sb + Sa applied LDC (11.87 ± 1.65 MPa) veneer group but not significant to the Sb (11.47 ± 0.97 MPa), Tbc (11.37 ± 1.49 MPa), and Tbc + Sa (10.83 ± 1.47 MPa) treated LDC groups (*p* > 0.05).

**Fig. 2:**
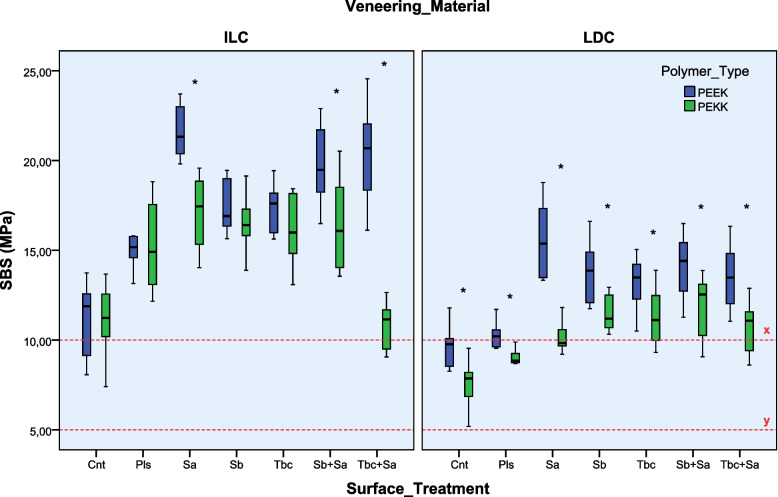
The box plot interval of test groups. The red hyphens show the lower limit of SBS as 5 MPa (y) and the clinically acceptable level of 10 MPa (x). * Indicates the significant differences between PEEK and PEKK groups with the same surface treatment and veneer material, according to the independent samples t-Test (*p* < 0.05)

The pairwise comparisons of mean SBS values of PEEK and PEKK groups, by the independent samples t-Test, showed that the PEEK groups were significantly higher than PEKK for Sa, Sb + Sa, and Tbc + Sa applied ILC veneer groups (*p* < 0.001) (Fig. 2). However, the SBS values of PEEK were significantly higher than PEKK polymer for all surface treatment applied LDC veneer groups (*p* < 0.05).

**Fig. 3:**
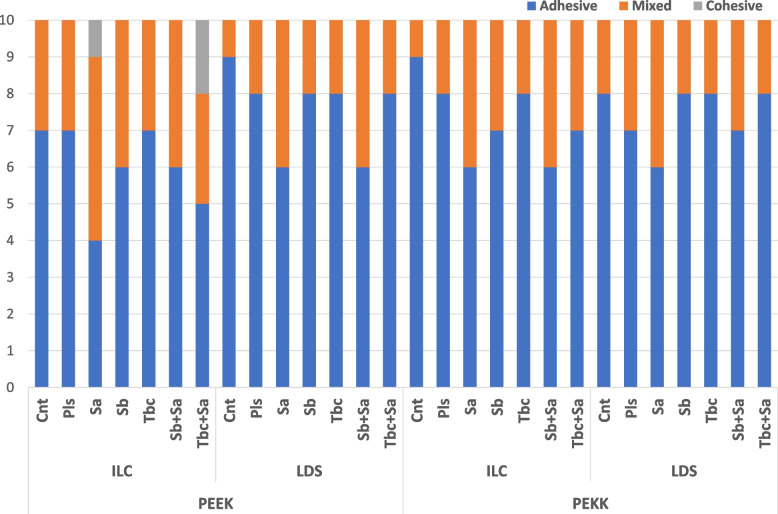
Failure mode distributions of test groups

No statistical difference was found among the failure mode results of the test groups by the Pearson chi-square test (*p* = 0.298). Considering the failure mode analyses, the common detected failures were adhesive for both PEEK (66.4%) and PEKK (74.6%) (Fig. 3). The cohesive failures were determined in PEEK polymer for Sa (10%) and Tbc + Sa (20%) applied ILC groups. A statistically significant correlation was detected between SBS and failure modes and indicated a moderate correlation between these two variables, according to the results of the Pearson correlation test (*p* < 0.001, r^2^ = 0.446).

**Fig. 4:**
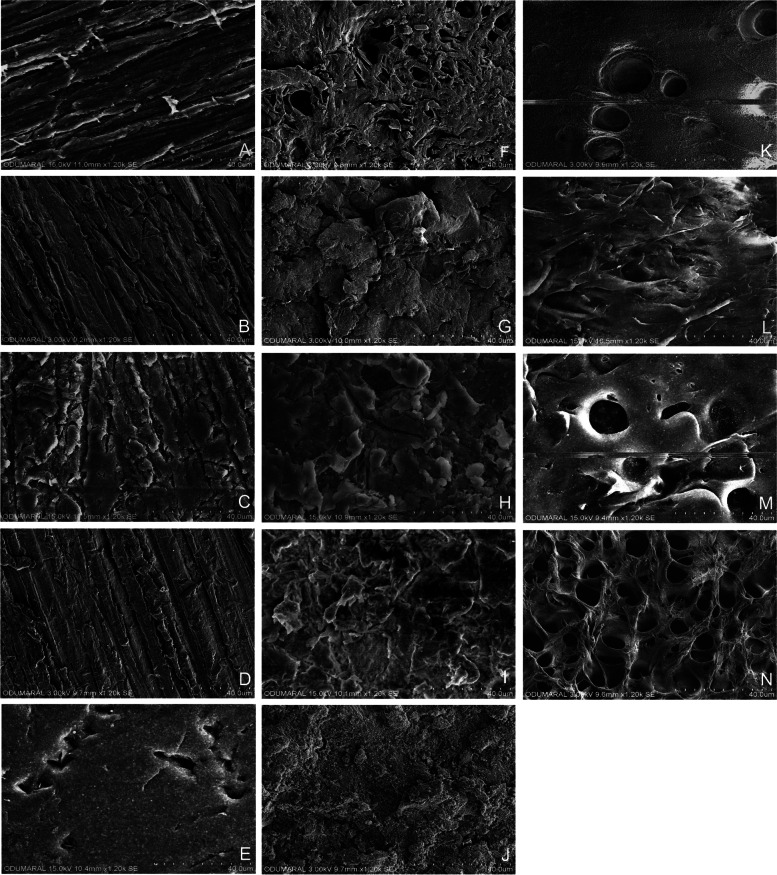
SEM images (× 1200) of PEEK and PEKK specimens: Untreated (Cnt) PEEK (**A**), PEKK (**B**); Plasma (Pls) applied to PEEK (**C**) and PEKK (**D**); 98% sulfuric acid (Sa) applied to PEEK (**E**) and PEKK (**F**); Sandblasted (Sb) PEEK (**G**) and PEKK (**H**); Tribochemical silica-coated (Tbc) PEEK (**I**) and PEKK (**J**); Sb + Sa applied PEEK (**K**) and PEKK (**L**); Tbc + Sa applied PEEK (**M**) and PEKK (**N**)

The SEM images of different surface treatments applied to PEEK and PEKK polymer specimens are shown in Fig. 4. The SEM images of both polymers Cnt and Pls groups were similar, with minor scratches on all the surfaces. Completely irregular voids and ridges surrounded by sharp stripes were determined on the SEM images of Sb and Tbc applied to both polymers. Uncommon deep grooves with demarcated borders were shown on the SEM image of Sa applied PEEK specimen in the rest of a smooth surface. Conversely, it has been demonstrated that Sa application predominantly affected all the surfaces of PEKK specimens with irregularities and an increased number of deep and small cavities. While the Sb + Sa applied specimen has a similar appearance to Sb, the Tbc + Sa applied specimen was reasonably different, with numerous, deeper, and larger holes, for PEKK polymer. The SEM images of Sa, Sb + Sa, and Tbc + Sa applied PEEK polymer specimens were quite similar.

## Discussion

It has been realized from the results of the present study that the surface treatment improved the SBS values of PAEKs to different veneering materials, and the type of polymer and veneering material varied the SBS results. Therefore, the null hypothesis of the present study was rejected.

Various mechanical, chemical, and combined surface treatments have been tested on the PAEK polymers to obtain clinically satisfactory bond strength results with veneering materials [2]. The bonding strength of PAEK polymers to LDC and ILC veneering materials was evaluated in the present study according to ISO 10477 standard [20, 23]. The SBS values of all test groups (7.57–21.55 MPa) were higher than the lower limit (5 MPa) of ISO 10477 standard and mostly higher than the clinically acceptable level of 10 MPa according to the related studies (Fig. 2) [8, 16, 20]. These results have shown that both veneering techniques with LDC or ILC materials would be suitable for PAEKs after tested surface treatments.

It has been reported in some previous studies that the 98% sulfuric acidic solution application ensured the highest SBS results for PEEK polymers compared to the other surface treatments [8, 11, 16, 17, 19]. Similarly, the most successful SBS results have been achieved for the 98% sulfuric acid solution application or its combinations with sandblasting and tribochemical silica coating techniques on PEEK polymer. Some related studies have explained the advanced results of sulfuric acid solution on the PEEK polymer: sulfuric acid solutions opened the aromatic polymer rings by charging onto the carbonyl and ether groups, thus increasing the surface polarity and wettability [2, 17, 19]. The surface of the sulfonated PEEK polymer contains reactive functional groups, which are responsible for the increased chemical bonding capacity with the resin-based adhesives [24]. On the other hand, 98% sulfuric acid solution application has also increased the SBS results of PEKK polymer. Still, it was not significantly higher than other surface treatment groups (*p* > 0.05). Previous studies confirmed that the 60 s of sulfuric acid solution (98%) application might improve the bonding performance of PEEK polymer to the resin-based material [2, 8, 13]. Still, comparable results could not be acquired for PEKK polymer [9]. Thus, it will be concluded from these results that 60 s of sulfuric acid solution (98%) etching is more efficient on the PEEK polymer than PEKK. Although the superior SBS results of 98% sulfuric acid application on PEEK polymer, a controversial situation has been detected on the SEM images with less irregular and smoother surfaces, compared to the PEKK groups (Fig. 4). The higher crystallinity and chemical resistance of PEEK polymer may lead to less influence of sulfuric acid solution with uncommon micro-grooves and lower surface irregularities, compared to the PEKK [21]. The higher ratio of ketone and carbonyl groups in the PEKK polymer structure may be responsible for sulfuric acid solutions' increased sensitivity and porosity [2, 9]. The effect of the concentration and duration of sulfuric acid solution on the bond strength and surface characteristics of PEKK polymer has been evaluated in a previous study, which declared that decreasing the concentration from 98 to 90% and duration from 60 to 5 s provided the highest bond strength results to composite resins with increased microcavities [9]. Further investigations are required to customize the concentration and duration of acidic solutions to gain better bonding results between the PEKK polymers and resin-based materials.

Sandblasting with Al_2_O_3_ and tribochemical silica coating with SiO_x_-Al_2_O_3_ particles are the other most recommended surface treatment techniques for PAEK polymers [3, 11, 16, 22]. The previous studies noticed that airborne-particle impact breaks the polymer shackles (C–C, C-H) and increases the surface roughness, thus improving the micro-mechanical bonding area and the wettability and penetration of the bonding agent inside of the polymer [11, 20, 24]. In addition, the released free radicals after the polymer chain disruption may also enhance the chemical linkage with the resin-based adhesives by provoking a chain transfer reaction with the adhesive agent [15, 24]. Therefore, the tribochemical silica coating technique has been applied to provide additional chemical modification on the polymer materials and the micro-mechanical alterations [6, 12, 25]. However, neither in related studies [11, 22] nor in the current study did the tribochemical silica coating with silica-modified 110 µm Al_2_O_3_ not statically improve the bonding performance of MMA-PETIA containing adhesive agent (Visio. link) to PAEK polymers than sandblasting with 50–110 µm Al_2_O_3_ (*p* > 0.05). The SEM images of the present study were consistent with these results that no further improvements were detected on the SEM images of Tbc implemented in both polymers, compared to the Sb groups (Fig. 4).

The combined application of Sb and Tbc after Sa treatment techniques has not been sufficiently investigated to modify PAEK polymers. The purpose of these combination groups was to create additional reactive functional groups by sulfonation reaction after the micro-mechanical air abrasion techniques. While the Tbc + Sa treatment caused significantly higher SBS values (20.48 ± 2.75 MPa) than the Tbc (17.41 ± 1.21 MPa) (*p* < 0.05), the Tbc + Sa and Sa (21.55 ± 1.45 MPa) groups were statistically not different (*p* > 0.05) for the ILC applied PEEK polymer groups. It will be concluded from these results that Tbc + Sa combined application is more advantageous than the single application of Tbc but insufficient than the single application of Sa. On the other hand, Tbc + Sa treatment caused significantly lower SBS values (10.84 ± 1.27 MPa) than not only the Tbc (16.19 ± 1.83 MPa) but also the Sa (16.56 ± 1.65 MPa) for the ILC applied PEKK polymer groups (*p* < 0.05). Similar results were reported in a previous study that the combined effect of sulfuric acid solution (98%) etching and tribochemical silica coating with 110 µm SiO_x_-Al_2_O_3_ was less efficient on the tensile bond strength (TBS) of ILC material to the PEKK polymer than the TBS values of their single application [3]. These unfavourable results will be concluded that Tbc + Sa combined application is not recommended to modify the PEKK polymer. The effect of Piranha solution for 30 s after sandblasting with 50 µm Al_2_O_3_ treatment on the TBS of ILC materials to the PEEK polymer has been evaluated in a related study [26]. In this study, while the combined application (19.9 ± 8 MPa) was significantly lower than the single application of Piranha solution (23.4 ± 9.9 MPa), it was higher than the air particle abrasion (16.5 ± 8 MPa) group (*p* < 0.05). Similarly, the single application of Sa caused better bonding strength than the combined practice of Sb + Sa in the present study (*p* < 0.05), but the Sb + Sa and Sb applications were statistically not different (*p* > 0.05) for the ILC-applied PEEK polymer groups. Controversially, the single application of Sa was significantly lower than the combined application of Sb + Sa for the LDC-applied PEKK polymer (*p* < 0.05), and the single and combined applied remaining test groups were statistically not different for the LDC applied both polymers (*p* > 0.05). Further investigations are required to customize the parameters of the combined application of S, Tbc after Sa treatment techniques onto both PAEK polymers and veneering materials.

Several novel surface treatments are also available for the PAEKs to eliminate the toxic features of the proven acid etching techniques and also the unfavourable results of sandblasting and tribochemical silica coating on the surface integrity of the polymers [2, 21, 27]. The concentrated sulfuric acid solutions are risky for both chairside and laboratory applications, not only with toxic features on human and tissue health but also may cause environmental pollution [5, 27]. The atmospheric cold plasma treatment with a solid, hand-operated, and portable plasma brush device is getting popular in dentistry to enhance moisture and surface-free energy by conducting the plasma energy onto the narrow application space [19]. The plasma application on a non-polar PAEK polymer surface improves as polar by creating functional groups containing oxygen, such as the carboxyl (C = O) and hydroxyl (-OH) radicals [15]. These functional groups trigger a reaction in the methacrylate-based adhesive systems to create a covalent bond between the adhesive and polymer [15, 22, 28]. In the present study, while the atmospheric cold plasma discharge was significantly improved the bond strength of ILC materials to the PAEK polymers (*p* < 0.05), no significant alteration detected for LDC groups (*p* > 0.05). In parallel to the result of the present study, atmospheric cold plasma application has been declared an effective surface treatment technique to improve the bond strength of resin-based materials to PAEK polymers [15, 28].

The SBS of two different veneering materials to the PAEKs was evaluated in the current study. Significant results were obtained for ILC veneering material, regardless of the surface treatment and polymer type. The bonding strength between existing framework materials (PEEK, gold-silver-palladium alloy, zirconia, and hybrid composite resin) and various resin-based cement materials (G-CEM Link Force, Panavia V5, RelyX Ultimate, Super-Bond C&B) have been evaluated in a related stud and declared that no satisfactory SBS results (0.6–0.8 MPa) obtained for PEEK-Panavia V5 groups without any bonding agent [29]. This controversial result will be concluded by using an MMA-PETIA content bonding material (Visio. link) in the current study that may be beneficial to improve the bond strength of the PAEK framework and veneer materials. It has been declared in a related study that identifying an adhesive system based on the composition is critical to the bonding performance of composite resins to PEEK polymer [22]. This study also stated that the chemical interaction of MMA-PETIA monomers with PAEK polymers was higher than other functional monomers based on the favourable TBS results of MMA-PETIA containing adhesive agent (Visio. link) for each pretreatment group (28.58–26.61 MPa). The UDMA-BDDMA content opaquer (Anaxblend Opaquer) application with the MMA-PETIA content bonding agent (Visio. link) will also be the reason for favourable SBS results of ILC veneer groups in the present study, which was recommended by previous studies to improve the bond strength of resin-based restoratives to PAEKs [10, 20, 21]. The fracture mode distributions of test groups in the current study are also coherent with the SBS results that the cohesive type of fractures was determined only for ILC veneer groups, and the number of adhesive type of fractures was more common for the LDC veneer groups (Fig. 3).

The effect of various surface treatments on the SBS of PEEK and PEKK polymers to ILC and LDC veneering materials has not been previously evaluated. However, the current study has several limitations; proven surface treatments in the current parameters like concentration, duration, or grain size are mostly recommended for PEEK polymer and must be customized for PEKK to gain better bonding results. A single type of bonding agent containing MMA-PETIA (Visio. link) has been used for all specimens. A variety of dual-polymerized composite resin cement containing Bis-GMA and TEGDMA with MDP monomer (Panavia V5) for the LDC specimens. In future studies, different types of adhesives, cement, polymers, veneering materials, and other variations of surface treatment procedures will be evaluated. The specimens of the present in-vitro study were hydrolytically aged, but the long-term dynamic-static or thermal aging process should be performed in future studies. The last limitation of the present study is the topographic alterations after the surface treatments have been mentioned and interpreted with the SEM analyses. In future studies, measuring the surface roughness values of the treated surface with devices such as contact profilometers or atomic force microscopes will provide more apparent and precise results.

## Conclusions

The following conclusions can be drawn according to the results of the current study,

- i. The tested surface treatments are efficient on both veneer materials' SBS values of PAEKs. While the sandblasting with 110 μm Al_2_O_3_ and tribochemical silica coating with 110 μm silica-modified Al_2_O_3_ techniques may significantly improve the SBS values of both polymers, the sulfuric acid solution (98%) application ensures the highest SBS results in PEEK polymer for both veneer materials.
- ii. The combined sulfuric acid solution (98%) with sandblasting or tribochemical silica coating could not significantly increase the SBS values.
- iii. The polymer type and veneer material parameters are also effective on the SBS result; thus, the parameters of surface treatments should be more specified for the applied veneering material and polymer type.

## Data Availability

The datasets generated during and/or analyzed during the current study are available from the corresponding author upon reasonable request.
